# Comparative Studies of the Structural and Physicochemical Properties of the First Fullerene Derivative FD-C_60_ (Fullerenol) and Second Fullerene Derivate SD-C_60_ (3HFWC)

**DOI:** 10.3390/nano14050480

**Published:** 2024-03-06

**Authors:** Djuro Koruga, Ivana Stanković, Lidija Matija, Dietmar Kuhn, Bastian Christ, Sofia Dembski, Nenad Jevtić, Jelena Janać, Vladimir Pavlović, Bart De Wever

**Affiliations:** 1 NanoLab, Department of Biomedical Engineering, Faculty of Mechanical Engineering, University of Belgrade, 11220 Belgrade, Serbia; imileusnic@mas.bg.ac.rs (I.S.); lmatija@mas.bg.ac.rs (L.M.); 2 NanoWorld, 11043 Belgrade, Serbia; 3 LAUS GmbH, 67489 Kirrweiler, Germany; dietmar.kuhn@laus.group; 4 Fraunhofer, Institute for Silicate Research ISR, 97082 Würzburg, Germany; bastian.christ@isc.fraunhofer.de (B.C.); sofia.dembski@isc.fraunhofer.de (S.D.); 5 TFT Nano Centre LLC, 11010 Belgrade, Serbia; 6 TEM Laboratory, Faculty of Agriculture, University of Belgrade, 11000 Belgrade, Serbia; vlaver@agrif.bg.ac.rs; 7 Altexa Development, 98000 Monaco, Monaco

**Keywords:** fullerene C_60_, first derivate C_60_, second derivate C_60_, TEM, AFM/MFM, NMR, XRD, TGA/DTA-MS-FTIR, zeta potential, endothermic/exothermic properties

## Abstract

In order to maximally reduce the toxicity of fullerenol (the first derivative of C_60_, FD-C_60_), and increase its biomedical efficiency, the second derivative SD-C_60_ (3HFWC, Hyper-Harmonized Hydroxylated Fullerene Water Complex) was created. Several different methods were applied in the comparative characterization of FD-C_60_ and SD-C_60_ with the same OH groups in their core. FD-C_60_ as an individual structure was about 1.3 nm in size, while SD-C_60_ as an individual structure was 10–30 nm in size. Based on ten physicochemical methods and techniques, FD-C_60_ and SD-C_60_ were found to be two different substances in terms of size, structure, and physicochemical properties; FD-C_60_, at 100 °C, had endothermic characteristics, while SD-C_60_, at 133 °C, had exothermic characteristics; FD-C_60_ did not have water layers, while SD-C_60_ had water layers; the zeta potential of FD-C_60_ was −25.85 mV, while it was −43.29 mV for SD-C_60_. SD-C_60_ is a promising substance for use in cosmetics and pharmaceuticals.

## 1. Introduction

Since their discovery about 45 years ago, fullerenes and their physiochemical properties and applications have attracted the attention of researchers. This carbon-based nanomaterial, particularly fullerene C_60_, has captured the extra attention of scientists due to its symmetry and physicochemical properties. Fullerene C_60_, as a molecular crystal, was predicted by Osawa [1], synthesized and identified using mass spectroscopy by Kroto et al. [2], produced in gram quantities by Huffman et al. [3], and viewed at atomic resolution using a scanning tunneling microscope (STM) by Koruga et al. [4]. It has a high icosahedral symmetrical arrangement of carbon atoms, and their eigenvalues T_1g_, T_2g_, T_1u_, and T_2u_ have four Fibonacci values 1/2$\left(1+\sqrt{5\;}\right)$, $-1/2\left(1+\sqrt{5\;}\right)$, $1/2\left(1-\sqrt{5\;}\right)$, and $-1/2\left(1-\sqrt{5\;}\right)$, respectively, (Φ, −Φ, −ϕ, and ϕ, respectively, in Table S1). The rotation–vibration spectra of the C_60_ icosahedral molecule has been investigated [5], and its dual particle–wave properties have been experimentally discerned [6]. Since then, some biomolecules, such as clathrin, microtubules, etc., have been shown to have similar icosahedral symmetry properties [7,8] to SD-C_60_, which allows them to establish a resonant interaction with SD-C_60_. This is possible because the electronic and vibrational states of biomolecules are determined by their symmetry, and the vibrational modes of SD-C_60_ can be resonantly transmitted to biomolecules and influence them.

Fullerene C_60_ is a spherical molecular crystal with 60 carbon atoms distributed over the surface of the sphere in 12 pentagons and 20 hexagons [2]. Pentagons are energetically closed structures (diamagnetic), while hexagons are open–closed structures that “breathe” (paramagnetic) [9].

Due to its physical properties, based on symmetry, C_60_ is not soluble in water. It can become toxic when it comes under the influence of external factors, which lead to the opening of one of the two (C=C) bonds in the hexagons. The first fullerene derivative, FD-C_60_ (commercially named fullerenol C_60_(OH)_x_)), was synthesized by adding OH groups in order to achieve water solubility as well as to reduce possible toxicity [10]. Water solubility depends on the number of OH groups, which can be different; however, the stability of fullerenol has proven to be the best if it has 12, 24, 36, or 48 OH groups. Since the diameter of the C_60_ molecule, at the position of the carbon atom, is 0.71 nm, and the outer diameter is about 1 nm (due to *π*-electrons), the diameter of fullerenol is roughly 1.3 nm with the addition of the OH groups. It has been shown that the toxicity of C_60_ is reduced by 50% if OH groups are added [11]. Fullerene hydroxylation increases water solubility and affects how these nanoparticles interact with biological systems. It has been demonstrated that increasing fullerene water solubility through surface modification is related to significantly decreased toxicity. Specifically, this study observed decreased toxicity of hydroxylated fullerene compared to the cytotoxic effects of fullerene aggregates in human skin (HDP) and liver carcinoma (HepG2) cells [11]. Similarly, it was observed that hydroxylation decreases the toxic potential of fullerene in mouse L929 fibrosarcoma, rat C6 glioma, and U251 human glioma cell lines [12]. Additionally, hydroxylated fullerene induced apoptotic changes in the investigated cell lines, while fullerene C_60_ induced necrotic cell death. The distinct effects of pristine and modified fullerene originate from the different nanoparticle interactions with the intracellular metabolic pathways [11,12].

The beneficial effects of fullerenol are well documented. In human breast cancer cell lines, C_60_(OH)_22_ inhibited cancer cell growth and suppressed doxorubicin-induced cytotoxicity [13]. In a study by Jiao et al. [14], fullerenol C_60_(OH)_20_ showed antitumor and antimetastatic activity in an in vivo EMT-6 breast cancer metastasis model. The antitumor effect of fullerenol C_60_(OH)_20_ may be exerted through its effects on oxidative stress status, inhibition of the formation of angiogenesis factors, or through modulation of the immune profile [15].

However, fullerene hydroxylation did not provide the absolute absence of toxicity in living systems [16,17]. Additionally, the degree of fullerene hydroxylation affects toxicity [18,19]. Therefore, the physicochemical properties of fullerene derivatives are of prime importance for achieving optimal effects in living systems.

In order to further reduce its toxicity or even completely eliminate it, as well as to improve the transfer of the C_60_ vibrational modes to biological water and biomolecules, the second derivative of C_60_, SD-C_60_ (C_60_(OH)_36_@(H_2_O)_144-2528_), commercially named 3HFWC–Hyper-Harmonized Hydroxylated Fullerene Water Complex, was designed, synthesized and tested [20,21,22]. In reference [22], an explanation of the creation of stable water layers around FD-C_60_ (fullerenol) and obtaining SD-C_60_ (3HFWC) as three-dimensional Penrose tilings (3DPTs) is given. This was achieved by sequentially changing the angle between the hydrogen bonds in the water molecules according to the Fibonacci sequence of the Φ number, i.e., by the eigenvalues of T_1u_, T_2u_, T_1g_, T_2g_ of icosahedral symmetry. This was the first time the angle between the hydrogen bonds in the water molecules was sequentially changed according to the sequence of Φ (3/2 = 1.5, 5/3 = 1.66, 8/5 = 1.60, 13/8 = 1.625, 21/13 = 1.615, 34/21 = 1.619, 55/34 = 1.617, 89/55 = 1.618, 144/89 = 1.617…). The experimental results showed that the stability of this change in water layers was stable for at least three years. This new technology of packing water molecules into 3D Penrose tiles with icosahedral symmetry enabled water to exert its biophysical effect on biomolecules without the need for biochemical reactions. Therefore, this could be the beginning of a new direction in medicine, which we can call “water-based nanomedicine”.

Experimental results with SD-C_60_ (3HFWC) in the field of biomedicine, agriculture, and cosmetics have shown desirable effects. Specifically, effects on melanoma [22,23,24], an influence on Alzheimer’s disease [25], a reduction of pain and improved memory in mice [26], the formation of hydrogen peroxide in tomatoes, and an increase in lycopene and regulation on the flow of water from the extracellular space into the cell [27]. Most of the current methods and techniques in the fight against cancer are of a destructive nature, destroying diseased cells, and therefore also healthy ones that are in the immediate environment. The SD-C_60_-based method works differently. It was found that SD-C_60_ (3HFWC) realized its anti-melanoma action through initiation of cell reprogramming, demonstrating senescence establishment and a minor contribution to cell death. Our goal was to show that we had developed a substance different from fullerenol capable of reprogramming malignant cells rather than causing tumor cell death, which thus might have serious consequences on proliferation.

Additionally, the beneficial effects of skin hydration, synthesis of collagen and elastin, and biophysical properties of the human skin were demonstrated [28,29].

Bearing in mind the increasing interest for the application of both FD-C_60_ (fullerenol) [13,14,15,16,17,18,19,30,31,32,33,34,35,36,37,38] and SD-C_60_ (3HFWC) [22,23,24,25,26,27,28,29] in biomedicine and cosmetics, it is necessary to present a comparative physicochemical characterization of them. There are several studies that present the physicochemical characteristics of fullerenol [39,40,41,42], but since fullerenol can have a different number of OH groups (usually from 12 to 48), there can be significant differences in the properties of fullerenol. For this reason, this paper characterizes fullerenol (FD-C_60_), which is a precursor to the second derivative of C_60_, SD-C_60_ (3HFWC), i.e., with the same number of OH groups. In order to determine size, stability, exothermic and endothermic properties, spectral characteristics, and the existence of water shells (layers), research concerning FD-C_60_ and SD-C_60_ in solution and in a dry state was carried out in several laboratories across Europe, using devices such as AFM/MFM, TEM/STEM, ^13^C-NMR, ^1^H-NMR, GPC, ZetaPro, XRD, TGA/DTA, UV–vis–NIR, and FTIR spectrometers [43,44,45,46,47,48,49,50,51,52,53] The experiment design is given in the Section 2; it was performed in such a way that by characterizing both of these nanomaterials, their main physicochemical properties could be obtained. On this basis, the conclusions could be drawn about their similarities and differences. This complex comparative approach was applied for the first time to FD-C_60_ (fullerenol) and SD-C_60_ (3HFWC), and the data have been made available to the scientific community.

## 2. Materials and Methods

### 2.1. Samples Preparation

The fullerenol (C_60_(OH)_36_) or FD-C_60_, with a molecular weight of 1332 Da, a dust composition (yellow color), and a purity of 99.99% (as a precursor of SD-C_60_), was ordered in a dark bottle from Solaris Chem, Vaudreuil-Dorion, QC, Canada. It was stored in a dark room, with a humidity of 35 ± 2% and a temperature of 20 ± 2 °C.

The SD-C_60_ (C_60_(OH)_36_)@(H_2_O)_144-2528_) or 3HFWC, with a molecular weight of 3.826–47.124 Da, was synthesized at the TFT NanoCenter, Belgrade, Serbia (3 g of fullerol was mixed with 20 L of ultra-pure water for commercial use), according to the patented procedure [20,21]. The formation of SD-C_60_ began with 0.150 g/L of hydroxylated fullerene C_60_(OH)_36_ dissolved in high-purity water (0.05 µS/cm) under the influence of an external oscillatory magnetic field +250/−92 mT according to the icosahedral eigenvalues T_1g_, T_2g_, T_1u_, and T_2u_ (Fibonacci numbers Φ, −Φ, −ϕ, and ϕ). At the same time, under the internal action of the vibrations of the C_60_ molecules (same vibration law as an external magnetic field) in a reactor at 37 °C, the formation of 3HFWC was realized [22]. The composition of SD-C_60_ (3HFWC) was as follows: (1) solid-state of 2.5–3%, (2) ordered water in chains (crystalline, linear chains between the solid-state 3HFWC units) 58–60%, (3) fullerenol (around which water layers failed to form) about 0.05%, and (4) free water about 38% [22]. The SD-C_60_ solid-state of 2.5–3% was the dry residue value obtained from the solution when dried immediately before the characterization experiments. The SD-C_60_ was stored in a dark bottle, in a dark place, at a room humidity of 35 ± 2% and a temperature of 20 ± 2 °C. In relation to when the SD-C_60_ solutions were produced, there were three solution samples: three years old, two years old, and eight months old. Investigations of SD-C_60_ in solution were carried out using UV–vis–NIR, FTIR, ZetaPro (Doncaster, UK), and GPC techniques, while dried SD-C_60_ (solid-state) was investigated using TEM/STEM, AFM/MFM, XRD, TGA/DTA-MS-FTIR and NMR techniques. Dry samples of SD-C_60_ were made immediately before the experiment; it was also recorded how long the solid form of SD-C_60_ could survive in solution (Table S2).

### 2.2. UV–vis–NIR and FTIR

UV–vis–NIR characterization of both FD-C_60_ (fullerol) and SD-C60 (3HFWC) was performed using a Lambda 500 spectrometer, Perkin-Elmer, USA, in the range of 250–3000 nm. FTIR characterization of both FD-C_60_ (fullerol) and SD-C60 (3HFWC) was performed using the Spectrum Spotlight 400 FTIR Imaging System, Perkin-Elmer, Waltham, MA, USA, in the range of 2500–14,000 nm. Additionally, the following instruments were used for the characterization: near-infrared spectrometer Lambda 1050+, Perkin Elmer, up to 2600 nm; Infrared Spectrometer Spectrum Two FT-IR, Perkin Elmer; Laser HeNe Class 1, with a scanned range of 370–7800 cm^−^^1^; UV–vis spectrometer Specord 205, Analytik Jena (Jena, Germany), with a resolution of ±0.5 nm and a scanned range of 190–1100 nm.

### 2.3. TEM and HAADF-STEM

Transmission electron microscopy (TEM) analysis of the dry particles of both FD-C_60_ (fullerenol) and SD-C_60_ (3HFWC) was performed in order to determine their solid-state size. The samples in a liquid state were applied to the TEM copper mash coated with carbon and dried in air. After drying the samples, they were analyzed and recorded in three TEM laboratories: (1) the CM12 Philips/FEI Transmission Electron Microscopy, Eindhoven, the Netherlands, magnification ×45,000 and ×60,000 (solution was 6 months old); (2) TEM, JEM 1400, JEOL, Tokyo, Japan, magnification ×120,000 up to ×200,000 (solutions were 8 months old and 3 years old); and (3) High-Angle Annular Dark Field Scanning Transmission Electron Microscopy (HAADF-STEM), Thermo-Fisher Talos/Osiris 200 kV (Waltham, MA, USA), Zeis Libra (Oberkochen, Germany) 120 kV, 200 kV electron energy, STEM mode with bright field, with evaluation software Thermo-Fisher Velox 3.7 and energy-dispersive X-ray analysis EDXS mapping = ChemiSTEM, with Z > 8 installed (the solution sample was 8 months old).

### 2.4. AFM/MFM

AFM/MFM characterization of FD-C_60_ and SD-C_60_ was performed after drying the samples on copper coated with carbon; the samples (solution was 8 months old) were stored in dark, closed containers and kept at room temperature until use. Characterization of the dry samples was performed using a JSPM-5200, Scanning Probe Microscope, JEOL, Tokyo, Japan. Two methods were used: AFM (atomic force microscopy) and MFM (magnetic force microscopy). Both techniques are non-invasive methods: the AFM method is based on van der Waals forces and London-type dispersive forces between the tip and sample; while MFM, in the non-contact imaging mode, is based on magnetic dipole–dipole interactions between the tip and sample (measuring ± deflection of the tip “ϕ” in degrees). For the purpose of investigating the magnetic gradient, specialized cantilevers, type HQ NSC18/Co-Cr Al BS (Micro Mash, Tallinn, Estonia), with a force constant between 1.2 and 5.5 N/m and a resonant frequency range from 60 to 90 kHz were used. Scan sizes at 30 nm, 100 nm, and 300 nm were performed.

### 2.5. XRD

Powder X-ray diffractograms (XEDs) were recorded on a Rigaku (Woodlands, TX, USA) SmartLab 3 kW from 5 to 85° 2Theta at room temperature. For preparation, an aqueous 3HFWC solution was lyophilized for 7 days to give a brownish powder. Fullerenol was used as a powder, as delivered from Solaris Chem, Canada.

### 2.6. TGA/DTA-MS-FTIR

TGA/DTA-MS-FTIR measurements were performed on an STA 449C Jupiter (TGA/DTA) and an Aeolos QMS 403 C (MS) from Netzsch, Germany, and a Bruker Tensor 27 (Billerica, MA, USA) with a gas chamber (FTIR). Lyophilized 3HFWC and fullerenol dried under vacuum (10 mbar/30 °C) were weighed in Al_2_O_3_ crucibles. During the measurements, the samples were heated from 30 to 900 °C with a constant heating rate of 10 K/min under a synthetic air atmosphere.

### 2.7. ^13^C-NMR and ^1^H-NMR

The Avance DRX 400, Bruker, BioSpin GmbH Cryo, at frequencies of 400 MHz (^1^H) and 100 MHz (^13^C), and a temperature of 300 K, was used with a solution-saturated solution. The sample was dissolved in D_2_O, and ^1^H and ^13^C spectra were recorded. ^1^H-NMR spectra were recorded with water suspension, while ^13^C-NMR spectra were recorded with water suspension without TMS.

### 2.8. Zeta Potential

The zeta potential was recorded via electrophoretic light scattering using the BeNano 180 ZetaPro analytical instrument. Sample preparation was as follows: the roller mixer was set at a measuring temperature of 25 °C, cell measurements were performed using the folded capillary cell, with five repetitions of the fold measurements and evaluation of the mean value from five measurements.

### 2.9. GPC

For gel permeation chromatography, we used the following: precolumn PSS Suprema: 5 µm, Guard, ID 8.0 mm × 50 mm; PSS Suprema: 5 µm, 30 Å, ID 8.0 mm × 300 mm PSS Suprema: 5 µm, 1000 Å, ID 8.0 mm × 300 mm; PSS Suprema: 5 µm, 1000 Å, ID 8.0 mm × 300 mm; Pump PSS SECcurity 1260 HPLC-pump, Agilent, Burladingen, Germany: flow rate of 1.0 mL/min, injection volume of 100 µL, and temperature 35 °C; detectors PSS SECcurity refractive detector (RI); PSS SECcurity ultraviolet light detector (UV); and Calculation PSS-WinGPC Unity Version 8.4. Sample preparation for gel permeation chromatography (GPC) was based on a procedure where the received sample solutions were transferred into the injections vials and injected by an autosampler without any further pre-treatment. Several pullulan standards with different molecular weights were measured to construct a calibration curve. The calculation of the average molecular weights and the molecular weight distribution of the samples was performed using the so-called “slice-by-slice” method based on the pullulan calibration.

## 3. Results

Since two spectroscopic domains, 2500–3300 nm and 6000–7000 nm, were important for fullerenol and 3HFWC application in biomedicine, we begin with the presentation of the similarities and differences between them from our results. The first spectral domain is important for the organization of water molecules into water layers (shells), while the second domain is important for the biophysical influence of the first and second C_60_ derivatives on biomolecules. Beside water (…H-O-H…O-H…O-H…) and DNA (A=T, C≡G), the amide-I (…H-N-C=O…H-N-C=O…) hydrogen bonds have a very important role in the secondary structure of biomolecules (proteins), their stability, conformation states, and functionality.

### 3.1. NIR and FTIR Spectroscopy

**Figure 1:**
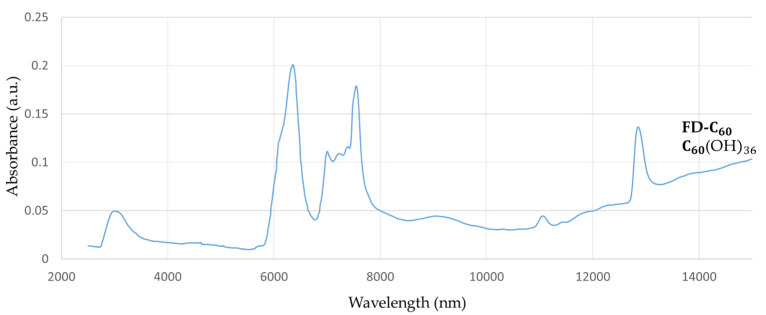
The FTIR spectrum of fullerenol (FD-C_60_) in the 2500–15,000 nm domain. There are four dominant peaks, one at 3088 nm (spontaneously water shell around FD-C_60_) with an intensity of 0.05 a.u., one at 6377 nm with an intensity of 0.20 a.u., one at 7562 nm with an intensity of 0.17 a.u., and one at 12,865 nm with an intensity of 0.13 a.u.

FD-C_60_ (fullerenol with an average of 36 OH groups, Figure S1) had one peak at 3088 nm with low intensity (0.05 a.u.), which means that it contained OH groups and had hydrogen bonds connected to water molecules from the humidity level (Figure 1). There were also three more peaks at 6377 nm, 7562 nm, and 12,865 nm with peaks intensity of 0.20, 0.17, and 0.13 a.u., respectively, that could affect biomolecules via their vibrational modes.

**Figure 2:**
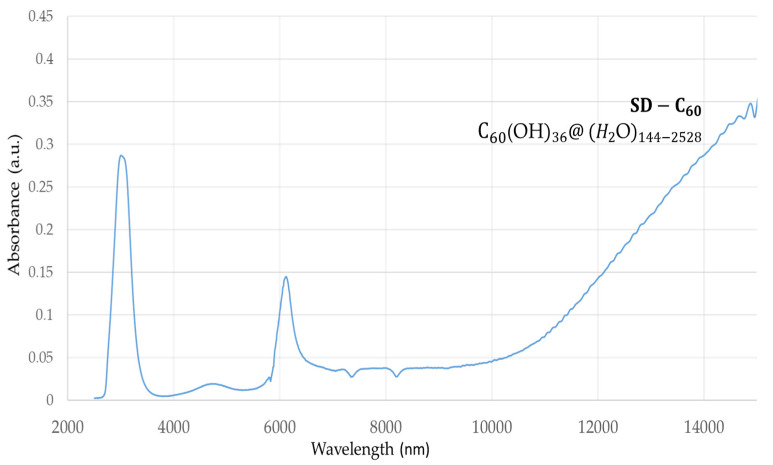
FTIR spectrum of 3HFWC (SD-C_60_) in the 2500–16,000 nm domain. There are two dominant peaks at 3064 nm (intramolecular “chelate” hydrogen bonds) with an intensity of 0.28 a.u., and 6132 nm with an intensity of 0.14 a.u. Furthermore, the spectrum exhibits an increasing value of 0.05–0.35 a.u. in the 10,000–15,000 nm domain, with a peak in this area above 15,000 nm (could not be detected because it was outside the instrument’s wavelength domain).

SD-C_60_ (3HFWC, whose precursor is fullerenol with 36 OH groups) had a peak at 3064 nm with an intensity of 0.28 a.u., which showed that due to the OH groups, water from the humidity level also had water layers (Figure 2). In addition, there was a peak at 6132 nm with an intensity of 0.14 a.u., and an elevated area from 10,000 nm to 15,000 nm with intensities of 0.05 a.u. and 0.35 a.u., respectively.

If the peaks from Figure 1 and Figure 2 are compared at around 3000 nm, it can be seen that the peak with 3HFWC was five to six times more intense than with fullerenol. This result shows that there are many more hydrogen bonds in 3HFWC than in fullerenol. Concerning the nature of the intramolecular water hydrogen bonds, they first formed rings on the surface of the sphere (“chelate”) and then ordered themselves into a closed water shell as three-dimensional Penrose tiles [22]. The C_60_(OH)_n_ replaced the ion role (in the classical sense) in the center of the coordinate bonds; this is the reason why “chelate” is written in quotation marks. As fullerenol (C_60_(OH)_n_) is a precursor of 3HFWC, when we subtracted the intensity of the fullerenol peaks from the 3HFWC peaks, we obtained the peak intensities of only the hydrogen bonding water shells (layers) of 3HFWC, which was 0.23 a.u. Additionally, a big difference in the peak intensity of about 0.22 a.u. was within the 10,000–15,000 nm range, which gives a two to seven times higher intensity value of the effect on biomolecules.

### 3.2. ^13^C-NMR and ^1^H-NMR

**Figure 3:**
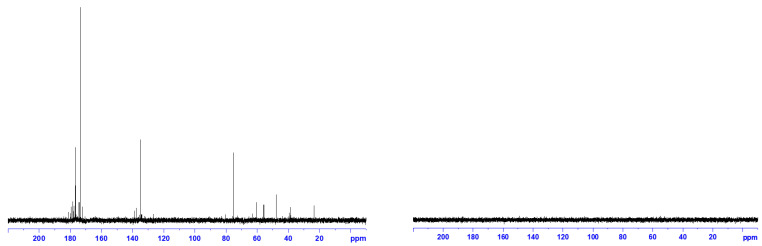
(**Left**) ^13^C-NMR spectrum with a water suspension of fullerenol (FD-C_60_) at a concentration of 0.15 μg/μL. Thirteen peaks from 172 to 181 ppm show the different forms of fullerenol (from 30 to 50 OH groups), with a dominant peak at 176.49 ppm (36 OH groups). The second peak from 126 to 138 ppm show C=C bonding in C_60_, while the peak at 75.25 ppm represents a C–OH group in the hydroxylated fullerene. (**Right**) ^13^C-NMR spectrum with a water suspension of 3HFWC (SD-C_60_) at the same concentration of fullerenol (0.15 μg/μL, as a precursor). ^13^C-NMR could not detect peaks of ^13^C atoms despite using the same concentration as in the fullerenol experiments. We assume that there are two possible reasons for this: (1) too few ^13^C atoms in the sample too detect a signal, or (2) the water layers of 3HFWC absorbed the magnetic signal of ^13^C atoms.

**Figure 4:**
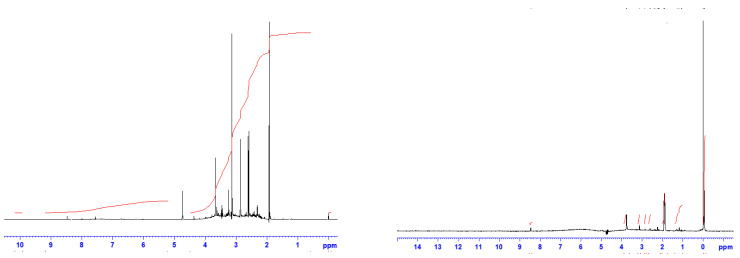
^1^H-NMR spectra of fullerenol (FD-C_60_) with six dominant peaks from 4.8 ppm to 1.9 ppm (**left**), and 3HFWC (SD-C_60_) with three dominant peaks from 0 ppm to 3.8 ppm (**right**). The black lines represent a chemical shift (ppm), while the red lines represent the integral spectra.

The characterization results of fullerenol and 3HFWC using ^13^C-NMR and ^1^H-NMR showed similarities and differences between these two substances (Figure 3 and Figure 4). The ^13^C-NMR spectrum of fullerenol had peaks from 172 to 181 ppm (the number of OH groups were 32, 36, 44, and 48, with a dominant peak at 176.49 ppm, i.e., 36 OH groups); signals (126–138 ppm) showing C=C bonds in the C_60_ molecule and C-O-H bonds (75,25 ppm) were observed. Having in mind that the intensity for 36 –OH groups was much higher, it can be concluded that the most abundant number of –OH groups was 36 (Figure S3).

As can be seen, no single peak was observed in the ^13^C-NMR spectrum of 3HFWC. This indicates two possibilities; either the ^13^C signal was so weak (in nature, only 1.1% of carbon atoms are ^13^C) that the device could not detect it or the 3HFWC water layers absorbed the ^13^C magnetic signal. Bearing in mind that the same fullerenol material was used in both cases at the same concentration (0.150 g/L), we are more inclined to think that the ^13^C signal was absorbed by the water layers rather than not being detected due to weakness. However, in order to provide a valid conclusion, it is necessary to do additional research.

^1^The H-NMR spectra, both ppm and integral, of fullerenol and 3HFWC without TMS are presented in Figure 4 and Table 1.

A significant chemical shift was seen in the 3HFWC ^1^H-NMR spectrum (without TMS) at 8.45 ppm, while fullerenol had many peaks between 8.76 and 4.81 ppm. The integral spectrum of 3HFWC had a value 3.57, while fullerenol had a value of 15.31. These structures and values tell us that protons in 3HFWC have a higher symmetrical order than fullerenol (Figure S4).

### 3.3. TEM Images

TEM images of dry fullerenol and dry 3HFWC were taken in three independent laboratories. Samples of the 3HFWC solutions, before drying, and from eight months to three years old, were sent. Information regarding the age of the solution concerned the stability of 3HFWC (soft solid state, which becomes a dry residue during drying) in the given solution. Fullerenol was prepared as a solution and dried just before the experiments.

**Figure 5:**
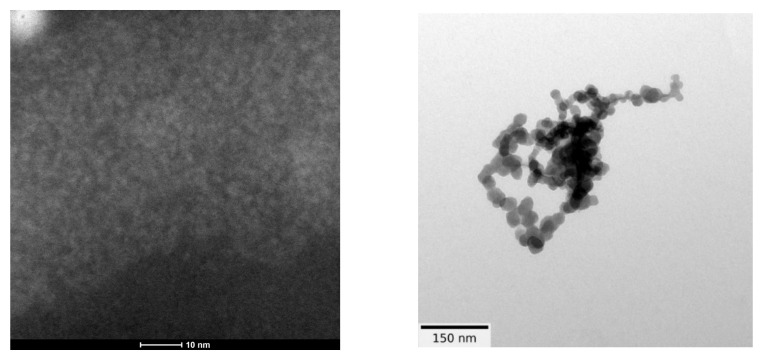
TEM images of dry fullerenol (FD-C_60_) (**left**) and 3HFWC (SD-C_60_) (**right**). The fullerenol molecule is about 1.3 nm in diameter, but under humidity, it may form agglomerates of different sizes (until hundreds nm) and shapes (linear, random foling, and balls). 3HFWC is a spherical molecule, about 15 nm in diameter (from 10 nm to 30 nm), and under humidity, it may form aggregates with dipole–dipole interactions.

TEM characterization showed that fullerenol had a diameter of 2.0 ± 0.6 nm (Figure 5, left) and could be clustered under humidity, forming large agglomerates (a few hundred nanometers) with a molecular weight between 1230 and 1536 Da (average 1332 Da). In theory, fullerenol is about 1.3 nm, which indicates that at room temperature and normal room humidity 35–45%, fullerenol is organized as a monomer (1.3 nm) or a dimer (2.6 nm).

**Figure 6:**
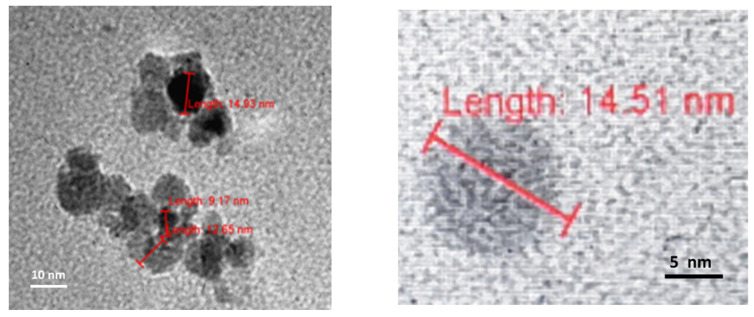
TEM images of the dry 3HFWC spherical molecule about 15 nm in size. In the center of the 3HFWC is fullerenol (diameter 1.3 nm) surrounded by water shells (layers, average diameter 14.51–1.30 = 13.21 nm). Water molecules are ordered according to icosahedral three-dimensional Penrose tiling (3DPT) [22].

**Figure 7:**
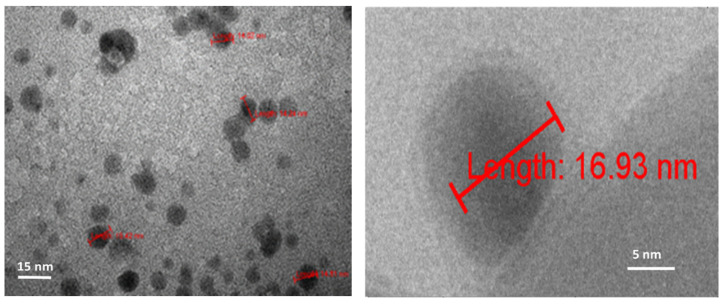
TEM images of dry 3HFWC spherical molecules about 17 nm in size. In the left image, the water shell has a dimeter of 15.63 nm (16.93–1.30 = 15.63 nm), with the water molecules ordered according to three-dimensional Penrose tiling (3DPT) [22].

All three TEM laboratories (two at the University of Belgrade and one at LAUS, Germany) obtained similar results for dry 3HFWC (SD-C_60_). Dry 3HFWC showed a spherical nanoparticle structure of sizes 10 nm–30 nm (Figure 5, right, and Figure 6 and Figure 7).

### 3.4. AFM/MFM Images

**Figure 8:**
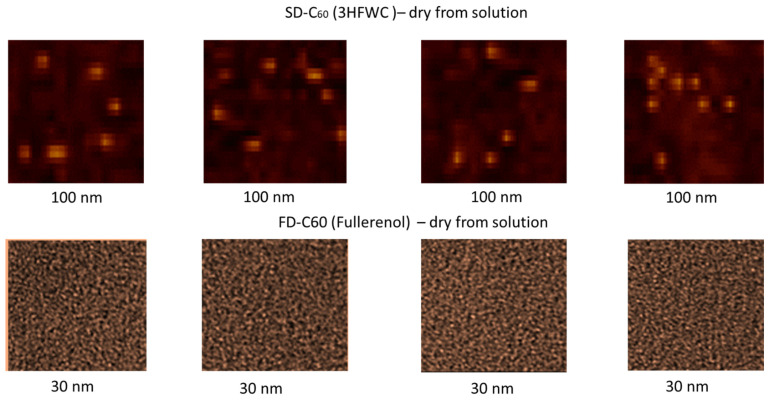
AFM/MFM images of dry 3HFWC (SD-C_60_) and dry fullerenol (FD-C_60_), with four different scanning spots. The scanning surface of 3HFWC was 100 × 100 nm, while for fullerenol it was 30 × 30 nm. Different scanning surfaces were chosen due to the different sizes of the two substances (fullerenol is about 1.3 nm, while 3HFWC is between 10 and 30 nm). 3HFWC molecules were observed as separate structures (individual), while in the images of fullerenol, individual molecules and smaller conglomerates were observed.

**Figure 9:**
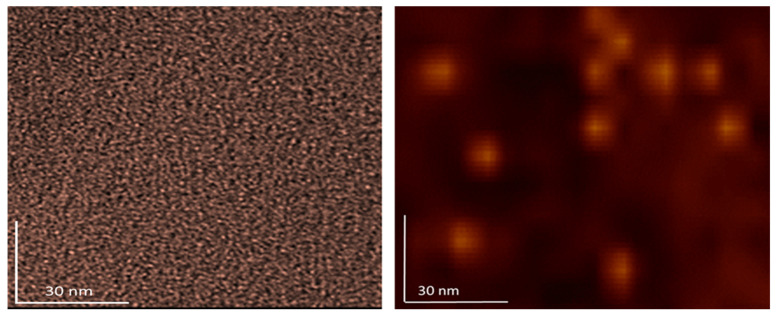
In order to clearly compare the sizes and organization of dry fullerenol (**left**) and dry 3HFWC (**right**), the AFM/MFM images of the same scanning spots, 100 × 100 nm, were taken. As can be seen from the pictures, the differences in size and organization are different. As fullerenol is a precursor to 3HFWC, they differ in their water layers, with thicknesses of 7 nm–14 nm forming around fullerenol.

**Figure 10:**
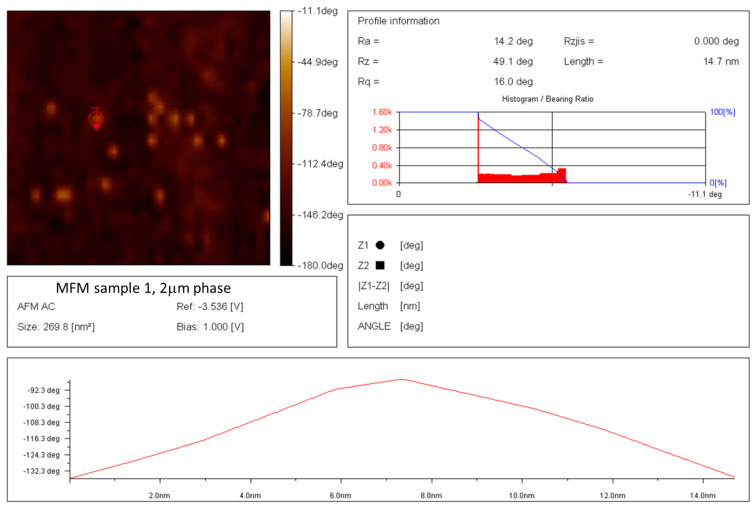
AFM/MFM image of dry 3HFWC with a scan area of 269.8 nm^2^, with 14 molecules of 3HFWC seen in the image. The molecular size of 3HFWC chosen for accurate measurement is 14.7 nm. As can be seen from the image, the surface of the molecules is not smooth (there are two subtle peaks at 5.9 nm and 7.4 nm), which is in agreement with previously obtained results [22].

**Figure 11:**
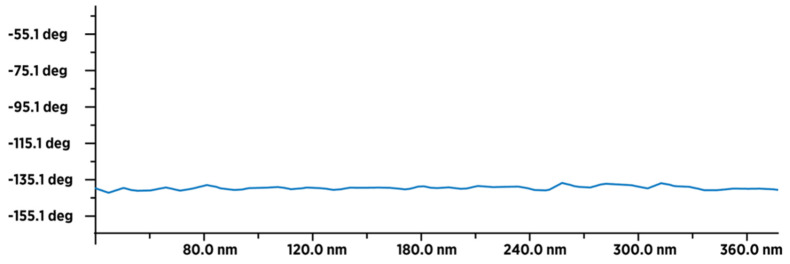
MFM image of 50–60 dry fullerenol molecules (in line) with an average type–sample interaction intensity of −140 degrees, which indicates that the sample is paramagnetic. The intensity and form of the graph indicate that a thin layer of humidity (water molecules) covered (evenly) the surface layer of fullerenol.

**Figure 12:**
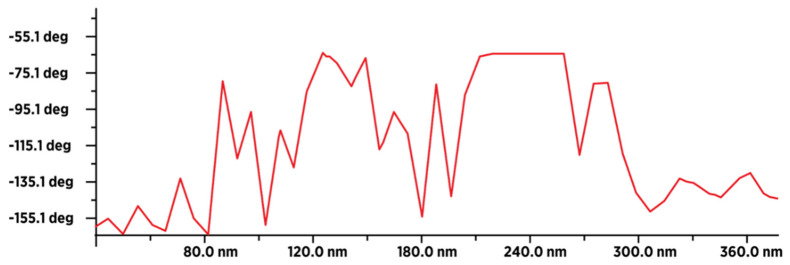
MFM image of about 18–20 dry 3HFWC molecules (in line) with an average type–sample interaction intensity of −100 degrees (minimum −165 degrees, and maximum −65 degrees), which indicates that the sample is less paramagnetic than fullerenol. This means that fullerenol, even if it has a smaller number of atoms (1.332 Da), has more unpaired electrons than 3HFWC, which has a significantly higher number of atoms (~3.200 Da). The intensity and form of the graph show that in addition to a thin layer of moisture, there are also water layers in 3HFWC (around fullerenol as the precursor to 3HFWC).

The results of AFM/MFM characterization of both dry substances, fullerenol, and 3HFWC, are presented in Figure 8, Figure 9, Figure 10, Figure 11 and Figure 12. Figure 8 shows four images of fullerenol and 3HFWC. The pictures clearly show the dimensions, shape, and number of particles. Fullerenol was most often organized as a dimer (size 2.6 nm, two molecules linked with water molecules from the humidity), while 3HFWC was seen as an individual particle with a size of about 15–30 nm.

High-resolution images of fullerenol and 3HFWC are shown in Figure 9. With fullerenol, in addition to dimers, organization into trimers could be seen, but in most cases, it formed a granular monomer structure. In the case of 3HFWC, spherical monomeric structures with a size of about 10–15 nm were clearly visible.

The exact value of 14.7 nm, one of several SD-C_60_ (3HFWC) molecules, is shown in Figure 10. A similar size of 3HFWC was obtained from several TEM images, which indicated that the most frequently recorded size of SD-C_60_ (3HFWC) was around 15 nm.

The result of a comparative examination of the presence of water in dried fullerenol (FD-C_60_) and dried 3HFWC (SD-C_60_) using MFM (magnetic force microscopy) showed that fullerenol contained a minimal number of water molecules originating from the humidity during the measurements (Figure 11), while 3HFWC had significantly more pronounced peak values and contained a significantly larger number of water molecules (Figure 12). The comparative presentation of the MFM values of fullerenol and 3HFWC on one diagram is shown in Figure S3; they are in agreement with previously published results [22].

### 3.5. X-ray Diffraction (XRD)

**Figure 13:**
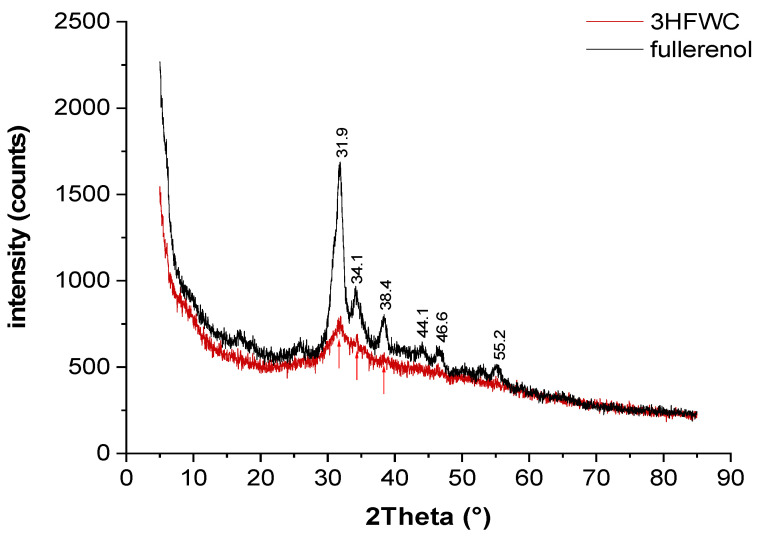
Powder XRD was performed to show the differences in the samples’ structural order. The XRD patterns of fullerenol show broad Bragg peaks at 27°–57° 2Theta due to partial crystallization of interstitial water. The higher water content (humidity + extra water) in 3HFWC induced a decrease in the reflex intensity and further broadened the reflexes.

Powder X-ray diffraction (XRD) was performed to see if there was a difference in the fullerenol and 3HFWC samples’ structural order (Figure 13).

Fullerenol was used as delivered (yellow dust material) and additionally dried under vacuum (10 mbar/30 °C); 3HFWC was lyophilized to a powder prior to the measurement.

Both fullerenol and 3HFWC are hydrophilic. Lyophilized fullerenol is a molecular powder with interstitial water clusters formed from humidity. The XRD patterns of fullerenol showed broad Bragg peaks at 27–57° 2Theta due to partial crystallization of interstitial water. The higher water content (humidity + extra water) in 3HFWC induced a decrease in the reflex intensity and further broadened the reflexes. This indicated that the structure of 3HFWC was less ordered due to extra water molecules organized in aperiodic three-dimensional Penrose tilings with different “lattice” sizes (there are sixteen different tiling types [22]). The Penrose three-dimensional tiling pattern (3DPT) is a type of quasicrystal, which means that it has an ordered yet never-repeating structure.

**Figure 14:**
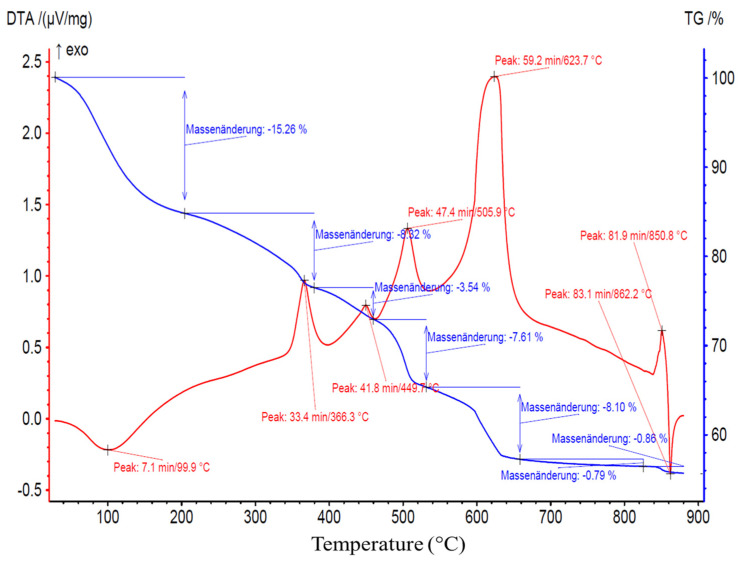
Fullerenol (FD-C_60_) TGA/DTA diagram with a peak at 99.9 °C (100 °C), thus indicating an endothermic reaction (water evaporation). Translation: Massenänderung—Mass change.

**Figure 15:**
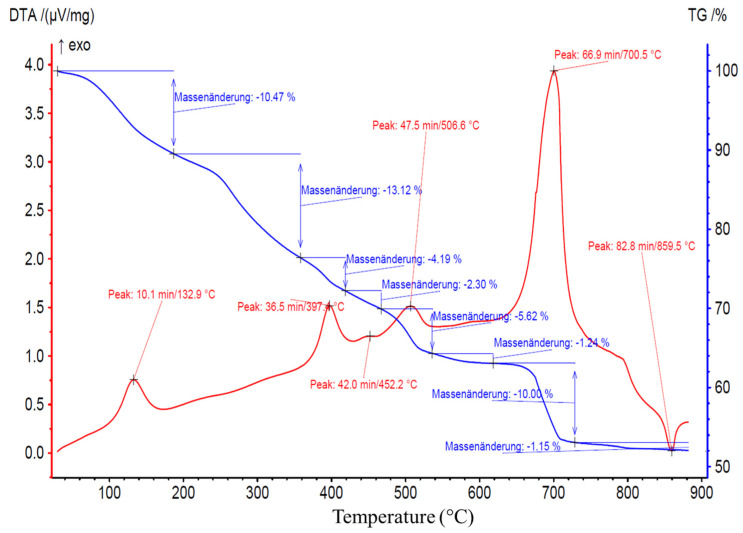
3HFWC (SD-C_60_) TGA/DTA diagram with a peak at 132.9 °C (133 °C), thus indicating an exothermic reaction (shifted to higher temperatures for evaporation). Translation: Massenänderung—Mass change.

However, it is precisely this organization that enabled SD-C_60_ (3HFWC) to have exothermic properties at a temperature of 133 °C, while fullerenol had endothermic properties at 100 °C (Figure 14 and Figure 15).

### 3.6. TGA/DTA-MS-FTIR

Figure 14 and Figure 15 show the results of the TGA/DTA measurements of fullerenol and 3HFWC, respectively. Since 3HFWC is proposed to be used as a cosmetic product, the focus in the interpretation of the results will be on temperature processes < 200 °C. In this temperature range, adsorbed solvents and water layers will be removed.

The mass loss of the fullerenol sample in the first mass loss step was 15.3 wt%. Taking into account that 3HFWC was freeze-dried at 0.37 mbar and 20 °C before measurement, it was noticeable that the mass loss up to approx. 200 °C was still high at 10.5 wt%. Interestingly, the corresponding DTA results of these processes show different enthalpies. While the first mass loss was an endothermic reaction in the fullerenol sample, 3HFWC showed an exothermic reaction. Additionally, the maximum of the DTA process was shifted to a higher temperature in 3HFWC. While the DTA peak in the endothermic process of fullerenol was at 100 °C, the maximum peak of 3HFWC was located at 133 °C.

TGA showed the mass loss of a sample during heating and sample decomposition, while DTA showed whether the single decomposition processes are of an endo- or exothermic nature. Exothermic reactions release energy, while endothermic processes, like the evaporation of solvents, need additional energy to be initiated. Thus, DTA measurements can indicate differences in the binding ratios of different substances during their decomposition.

Thermogravimetric analysis (TGA) and differential thermo analysis (DTA) coupled with mass spectrometry (MS) and infrared spectroscopy (FTIR) were performed on 3HFWC to identify any differences in the water layer bonding.

Both MS and FTIR demonstrated the release of water at temperatures below 200 °C under synthetic air conditions. 3HFWC released water in an exothermic reaction, while fullerenol released water in an endothermic reaction. Under the same sample treatment in a vacuum, differences in the reaction enthalpies, while releasing water, were obtained.

**Figure 16:**
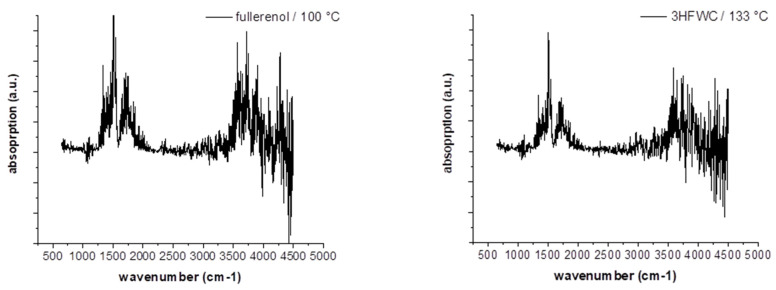
FTIR spectra of fullerenol (FD-C_60_) during the first endothermic DTA process at 100 °C (**left**) and 3HFWC (SD-C_60_) during the first exothermic DTA process at 133 °C (**right**) in the TGA/DTA-MS-FTIR investigation.

**Figure 17:**
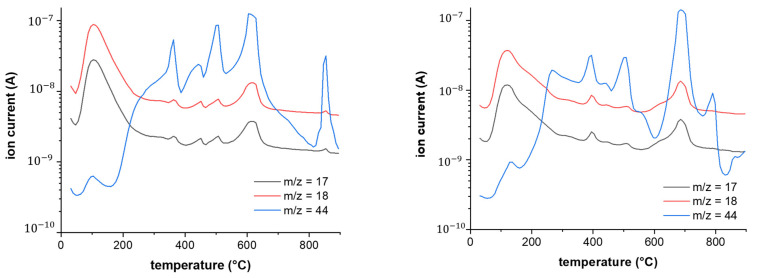
The MS spectra of fullerenol (FD-C_60_) at 100 °C (first endothermic DTA process) shows *m*/*z* fragments of 18 (H_2_O^+^) and 17 (OH^+^), while signals of fragment 44 (CO_2_^+^) are very low at 100 °C (**left**). The MS spectra of 3HFWC (SD-C_60_) at 133 °C (first exothermic DTA process) show *m*/*z* fragments of 18 (H_2_O^+^) and 17 (OH^+^), while signals of fragment 44 (CO_2_^+^) are very low at 133 °C (**right**).

Figure 16 shows the FTIR spectra of fullerenol at 100 °C and 3HFWC at 133 °C during the DTA signal’s maximum. In the case of MS (Figure 17), the *m*/*z* fragments, which can be detected below 200 °C—17 (OH^+^), 18 (H_2_O^+^), and 44 (CO_2_^+^)—were plotted.

FTIR only indicated a release of water, which was highlighted in the MS due to the high ion currents of *m*/*z* = 17 (OH^+^) and 18 (H_2_O^+^). CO_2_ (*m*/*z* = 44) was also released in small amounts, with an ion current of two potencies less (10^−9^ → 10^−7^).

The decomposition of 3HFWC and fullerenol at higher temperatures (<800 °C) took place in a similar way, with a tendency that the DTA signals for 3HFWC shifted to slightly higher temperatures. This indicated a structural difference in the two samples and differences in the water layers of 3HFWC, since even with the same pre-treatment, the differences in reaction enthalpies can be seen.

As no additional bands and *m*/*z* fragments were detected in fullerenol, water was also released from 3HFWC, and no further decomposition processes took place below 200 °C. Bearing in mind that 3HFWC released water in an exothermic reaction, water molecules were bonded to 3HFWC in a different way than in fullerenol.

Interestingly, the last decomposition process at 850–860 °C showed an endothermic decomposition process of 3HFWC but an exothermic followed by an endothermic process in fullerenol. This differences in decomposition behavior also indicated a structural difference in the samples.

### 3.7. GPC

**Figure 18:**
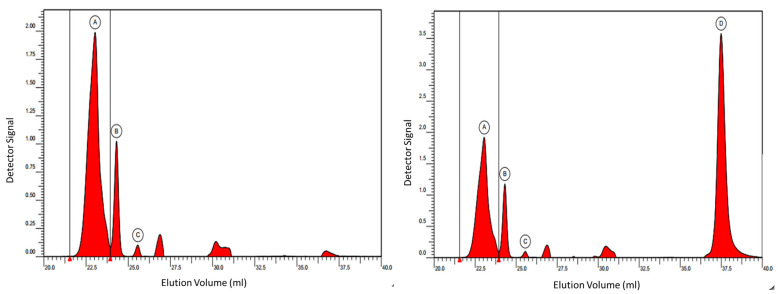
GPC fullerenol diagram (**left**) with three peaks (A~2.00 a.u., B~1.00 a.u. and C~0.10 a.u.), and GPC diagram of 3HFWC (**right**) with four peaks (A, B, C with the same intensity as fullerenol and a new peak D~3.5 a.u.). As can be seen, peak D intensity of 3HFWC (right) is dominant and much higher than peaks A, B, and C. Since fullerenol is a precursor for 3HFWC synthesis, peaks A, B, and C exhibit the same intensity. Peak D in this case may only represent the water layers of 3HFWC.

The gel permeation chromatography (GPC) results of fullerenol (FD-C_60_) and 3HFWC (SD-C_60_) and analysis of the UV signal at 250 nm are presented in Figure 18. Since fullerenol was expected to exhibit UV activity, calculations of the molar mass distributions were only carried out for UV-active species. The peak areas of the refractive index (RI) signal of side components were analyzed as well. The peaks at the elution volumes of approx. 27 and 31 mL were presumed to be system peaks.

A summary of the GPC results for 3HFWC were as follows: The peak area of fullerenol showed three different separate peaks, while 3HFWC showed four different separate peaks. The relative content of each “species” and the molar mass at the peak maximum, Mp, were different. Three peaks and their observed masses were within the calibration curve, while one (peak D) was out of the calibration curve. We assumed this was 3HFWC with water layers. The mass of the fourth peak (D) could not be determined, but it should be more hydrophilic than fullerenol.

### 3.8. Zeta Potential

At the same concentration of C_60_(OH)_36_ (0.150 g/L) in water (pH = 6.24, conductivity 2.2 μS/cm), the pH of the fullerenol (FD-C_60_) solution was 9.349, while the pH was 7.107 for the 3HFWC (SD-C_60_) solution (Table 2).

The zeta potential (ZP) value is a very important criterion for discerning the stability of a substance (Figure S7). The ZP of fullerenol was −25.85 mV, while for 3HFWC, it was −43.29 mV, thus indicating that 3HFWC is more stable than fullerenol. These findings clearly underline the difference between 3HFWC and its precursor (fullerenol) (Table 2).

The zeta potential (electrostatic charge) of 3HFWC was higher than that of fullerenol at an equivalent concentration. This was due to the hydration shells of water surrounding the fullerenol molecules having more ordered bonding and, thus, greater polarity. This explains the stability of 3HFWC, possessing a greater electrostatic charge, greater electrostatic repulsion between 3HFWC molecules, and less agglomeration/sinking.

## 4. Discussion and Conclusions

Characterization of fullerenol (FD-C_60_) and 3HFWC (SD-C_60_) in solution at a temperature of 20 ± 1 °C using NIR and FTIR spectroscopy showed differences in the spectra between the two substances within the 2500–3300 nm (NIR) and 2500–14,000 nm (FTIR) ranges. Using NIR spectroscopy, in addition to atmospheric pressure and a temperature of 20 °C, for 3HFWC (SD-C_60_), spectra were run at temperatures of 80 °C, 90 °C, 120 °C, and 140 °C (Figure S8). The obtained results indicated that at temperatures between 120 °C and 140 °C, the water layers evaporate, i.e., the non-covalent hydrogen bonds are broken. The exact temperature that hydrogen bond breaking occurs was identified by the TGA/DTA tests. For 3HFMC, this happened at a temperature of 132.9 °C (133 °C), while for fullerenol, evaporation (moisture) happened at 100 °C.

^13^C-NMR and ^1^H-NMR showed clear structural differences of fullerenol and 3HFWC. The absence of a peak on the ^13^C-NMR spectrum of 3HFWC could be interpreted in two ways. It may have been a weak signal (because only 1.1% of carbon atoms in nature are ^13^C); however, bearing in mind that we obtained clean signals with the same material in the form of fullerenol, we suspect that it is possible that the water layers of 3HFWC may have absorbed the ^13^C signal. Since the difference in frequency between the water layers and ^13^C signal is of the order of 6 (10^6^), this opinion is based on the consideration of coupling at a high-frequency mode (O-H and O…H in water layers of 3HFWC, range ~10^14^ Hz) and a low-frequency mode (resonant Larmor frequency for ^13^C, range 10^8^ Hz) through their modulation of the amplitudes and phases. The general approach of nonlinear coupling (theoretical and experimental) of high- and low-frequency modes has been explained in the literature [54,55,56,57,58,59,60,61,62].

Additionally, the comparison of the FTIR signal intensity for C_60_, fullerenol, and 3HFWC (Table 3) indicated that the vibrational peaks of C_60_ (with the corresponding shift) were present in both fullerenol and 3HFWC; however, there was a big difference of up to seven times greater when it came to 3HFWC. The difference in the peak intensity between C_60_ and fullerenol was almost non-existent. To confirm the absence of a ^13^C-NMR signal for 3HFWC and explain the differences in the FTIR spectra of C_60_, fullerenol, and 3HFWC, additional extensive research is needed, which we intend to carry out in the near future.

Characterization of the dry 3HFWC substance using TEM was performed three times, in 2018, 2019, and 2023, in three independent laboratories (Supplementary Materials Table S2). The age of the substance ranged from several months to four years. In all cases, it was shown that the size of dry 3HFWC was on average 15 nm depending on the number of water layers it contained. The characterization of fullerenol was performed only once in 2023, and it was shown that the size was between 1.3 and 2.6 nm; in some cases, it was around 1 μm, which indicates that as soon as it comes into contact with moisture from the air it forms aggregates. Similar results were obtained with AFM/MFM characterization. 3HFWC was observed as a one-body object of size 10–30 nm. Fullerenol as a one-body object was 1.2–1.4 nm and from 2.5 to 6.0 nm as aggregates. The magnetic force microscopy spectra of 3HFWC and fullerenol were different. Since the intensity of the magnetic spectra is proportional to number of dipole–dipole interactions, it means that 3HFWC is richer with molecules which possess dipoles (water in layers) than fullerenol (only water from humidity). The possibility of characterizing various types of materials at the nano level, including biomaterials, using MFM has been reviewed in ref. [63,64,65,66,67,68].

Stability and pH values are important for substances that are used in biomedicine and cosmetics. The zeta potential of the 3HFWC substance was −43.29 mV, which indicates that it is very stable, while fullerenol had a value of −25.85 mV, which indicates that it is at the limit of its stability. Additionally, there was a difference in pH values, with 9.35 for fullerenol and 7.11 for 3HFWC.

In this paper, a contradiction regarding the degree of the structural order of fullerenol and 3HFWC obtained using ^1^H-NMR and XRD was explained. When the order of protons in the structure was observed, 3HFWC was more ordered than fullerenol. The reason for this lies in the possibility of O-H rotation in the C-O-H structure, thus permitting the hydrogen atom to occupy various positions. Bearing in mind that it possesses about 36 OH groups, the positions of the hydrogen atoms form a very complex system. However, with 3HFWC, this is not the case because all the hydrogen atoms in the precursor (fullerenol) and in the three-dimensional Penrose tiling (3DPT) are fixed. However, if these two structures are considered from the aspect of classical crystallography (XRD), then fullerenol has a more ordered structure than 3HFWC; this is because fullerenol is a molecular crystalline structure while 3HFWC is a quasi-crystalline molecular structure because 3DPT (in water layers) are aperiodically ordered.

The TGA/DTA-MS-FTIR measurements showed the release of water at temperatures below 200 °C under synthetic air conditions. There were differences in the bonding and release of the water layers. 3HFWC released water (humidity water + extra water) in an exothermic reaction; fullerenol released water (humidity water) in an endothermic reaction. Under the same sample treatment in a vacuum, the differences in the reaction enthalpies when releasing water were obtained.

SD-C_60_ (3HFWC: C_60_(OH)_36_@(H_2_O)_144-2252_ + humidity) showed a different decomposition behavior under a synthetic air atmosphere compared to FD-C_60_ (fullerenol: C_60_(OH)_36_ +humidity). The main differences were as follows: (1) 3HFWC showed an exothermic reaction at 133 °C when releasing water, while (2) fullerenol showed an endothermic release of water at 100 °C. With one exception, all the decomposition steps were slightly shifted to higher temperatures. The exception was the last exothermic process, which was shifted to lower temperatures for 3HFWC.

In summary, the core result was that water is bound in a different way in SD-C_60_ (3HFWC) than in FD-C_60_ (fullerenol), and fullerenol and 3HFWC are two different substances in terms of their structure, size, and physicochemical properties. The experimental results obtained in this research using various techniques (Section 2) showed that SD-C_60_ (3HFWC) is a substance with excellent potential application in both cosmetics and pharmaceuticals. This new active ingredient acts biophysically through the vibrational modes of the hydrogen bonds in water and biomolecules, thus significantly affecting the conformational states of biomolecules. If the conformational states of biomolecules are diverse, the vibrational effects of SD-C_60_ (3HFWC) will restore them to their normal original state.

Experiments on the application of fullerenol and 3HFWC in biomedicine have been reported, but there are no comparative studies in which the number of OH groups is the same in fullerenol (FD-C_60_) and 3HFWC (SD-C_60_). As in this paper, we plan to research both substances with the same number of OH groups, as this is the only way to be sure of the differences in their effects on biological systems in vitro and in vivo.

## Figures and Tables

**Table 1:** Differences between fullerenol and 3HFWC based on the ^1^H-NMR spectra (chemical shift/ppm and integral difference without TMS). The difference in the chemical shift is small, about 0.3 ppm, while the integral value is significantly different, about 4.3 times.

3HFWC without TMS Chemical Shift/ppm	3HFWC without TMS *Integral*	Fullerenol without TMS Chemical Shift/ppm	Fullerenol without TMS *Integral*
8.459	3.57	8.765–4.811	15.31

**Table 2:** Zeta potential, pH, electrophoretic mobility, and conductivity of fullerenol and 3HFWC (same concentration of C_60_(OH)_36_, 0.150 g/L, in both the fullerenol and 3HFWC solutions).

Test Substance	Temperature (°C)	pH	Zeta Potential (mV)	Electrophoretic Mobility (μm/s)/(Vcm)	Conductivity (μS/cm)
Fullerenol (FD-C_60_)	25	9.35	−25.85 ± 1.71	−2.01 ± 0.13	0.18
3HFWC (SD-C_60_)	25	7.11	−43.29 ± 1.23	−3.37 ± 0.10	0.17
Difference	0	2.24	−17.44	−1.36	0.01

**Table 3:** Wavelength shift and difference in the peak intensities of C_60_ [46], fullerenol (Figure 1) and 3HFWC (Figure 2) in the 7000–9000 nm FTIR range.

Peak		C_60_	Fullerenol	C_60_/Fullerenol (Shift/Difference)	3HFWC	C_60_/Fullerenol (Shift/Difference)
1.	Wavelength (nm)	7.003	6.370	633	7.340	337
	Intensity (a.u.)	0.192	0.190	0.002	0.027	0.165
2.	Wavelength (nm)	8.453	7.555	898	8.220	665
	Intensity (a.u.)	0.210	0.178	0.032	0.028	0.150

## Data Availability

Data are contained within the article and its Supplementary Materials.

## Supplementary Materials

The following supporting information can be downloaded at: https://www.mdpi.com/article/10.3390/nano14050480/s1, Table S1: Icosahedral symmetry group. Table S2: Characterization of dry 3HFWC in five independent institutions. Figure S1: Fullerenol OH group determination. Figure S2: Structural order of fullerenol and 3HFWC. Figure S3: Water presence in fullerenol and 3HFWC. Figure S4: Zeta potential of fullerenol and 3HFWC. Figure S5: NIR spectrum of 3HFWC at different temperatures. References [69,70,71,72] are cited in Supplementary Materials.
