# Supplementary material for: Comparative Studies of the Structural and Physicochemical Properties of the First Fullerene Derivative FD-C60 (Fullerenol) and Second Fullerene Derivate SD-C60 (3HFWC)
Source: Nanomaterials (Basel). 2024 Mar 6;14(5):480. doi: 10.3390/nano14050480 (PMC10935211; doi:10.3390/nano14050480)
Supplement: Supplementary file 1 [file nanomaterials-14-00480-s001.zip › nanomaterials-2879398-supplementary.pdf]

Supplementary Materials

Comparative Studies of Structural and Physicochemical Properties of the First Fullerene Derivative FD-C_{60} (Fullerenol) and the Second Fullerene Derivate SD-C_{60} (3HFWC)

Djuro Koruga [1,2,*], Ivana Stanković [1], Lidija Matija [1], Dietmar Kuhn [3], Bastian Christ [4], Sofia Dembski [4], Nenad Jevtić [5], Jelena Janać [5], Vladimir Pavlović [6] and Bart De Wever [7]

1 NanoLab, Department of Biomedical Engineering, Faculty of Mechanical Engineering, University of Belgrade, 11220 Belgrade, Serbia; imileusnic@mas.bg.ac.rs (I.S.); lmatija@mas.bg.ac.rs (L.M.)
2 NanoWorld, 11043 Belgrade, Serbia
3 LAUS GmbH, 67489 Kirrweiler, Germany; dietmar.kuhn@laus.group
4 Fraunhofer, Institute for Silicate Research ISR, 97082 Würzburg, Germany; bastian.christ@isc.fraunhofer.de (B.C.); sofia.dembski@isc.fraunhofer.de (S.D.)
5 TFT Nano Centre LLC, 11010 Belgrade, Serbia
6 TEM Laboratory, Faculty of Agriculture, University of Belgrade, 11000 Belgrade, Serbia; vlaver@agrif.bg.ac.rs
7 Altexa Development, 98000 Monaco, Monaco
* Correspondence: dkoruga@mas.bg.ac.rs or djuro.koruga@gmail.com

Table S1. Icosahedral symmetry group that determines the energy states (T_{1g}, T_{2g}, T_{1u} and T_{2u}) of structures and processes. Fibonacci numbers Φ, -Φ, ϕ, -ϕ are subset of icosahedral symmetry group (± ½(1+√5 and ± ½(1-√5)) (Adapt from Kettle, S.F.A., Symmetry and structure, John Willey and Sons, Chichester, 1995, Dresselhaus, M.S., Dresselhaus, M.S.,Eklund,P.C., Science of Fullerenes and Carbon Nanotubes, Academic Press, San Diego,1996. Icke, V., The force of symmetry, Cambridge University Press, Cambridge, 1995, Hargittai,I., Symmetry II: Unifying Human Understending, Pregamon Press, Oxford, 1989) [69–72].

I/I_h	E	$12C_5$	$12C_5^2$	$20C_3$	$15C_2$	i	$12S_{10}$	$12S_{10}^3$	$20S_6$	15σ	III	IV	
A_g	1	1	1	1	1	1	1	1	1	1		$x^2+y^2+z^2$	$\Phi = 1/2(1+\sqrt{5})$
T_{1g}	3	$\frac{1}{2}(1+\sqrt{5})$	$\frac{1}{2}(1-\sqrt{5})$	0	−1	3	$\frac{1}{2}(1-\sqrt{5})$	$\frac{1}{2}(1+\sqrt{5})$	0	−1	(R_x, R_y, R_z)		$-\Phi = -1/2(1+\sqrt{5})$
T_{2g}	3	$\frac{1}{2}(1-\sqrt{5})$	$\frac{1}{2}(1+\sqrt{5})$	0	−1	3	$\frac{1}{2}(1+\sqrt{5})$	$\frac{1}{2}(1-\sqrt{5})$	0	−1			$\phi = -1/2(1-\sqrt{5})$
G_g	4	−1	−1	1	0	4	−1	−1	1	0			$-\phi = 1/2(1-\sqrt{5})$
H_g	5	0	0	−1	1	5	0	0	−1	1		$(2z^2-x^2-y^2, x^2-y^2, xy, yz, zx)$	
A_u	1	1	1	1	1	−1	−1	−1	−1	−1			
T_{1u}	3	$\frac{1}{2}(1+\sqrt{5})$	$\frac{1}{2}(1-\sqrt{5})$	0	−1	−3	$-\frac{1}{2}(1-\sqrt{5})$	$-\frac{1}{2}(1+\sqrt{5})$	0	1	(x, y, z)		
T_{2u}	3	$\frac{1}{2}(1-\sqrt{5})$	$\frac{1}{2}(1+\sqrt{5})$	0	−1	−3	$-\frac{1}{2}(1+\sqrt{5})$	$-\frac{1}{2}(1-\sqrt{5})$	0	1			(x^3, y^3, z^3)
G_u	4	−1	−1	1	0	−4	1	1	−1	0			$[x(z^2-y^2), y(z^2-x^2), z(x^2-y^2), xyz]$
H_u	5	0	0	−1	1	−5	0	0	1	−1			

Table S2. Characterisation of dry 3HFWC in five independent institutions. SD-C_{60} (3HFWC) is produced as solution and dryed just before experiment. This information tell us how long soft-solid state of substance was stable (“old”) in solution (TFT – TFT Nano Centre, NW – NanoWorld).

No.	**SD-C_{60} (3HFWC) solution produced (NW/TFT)**	**SD-C_{60} (3HFWC) Dry (experiment done)**	**SD-C_{60} (3HFWC) Old (standing in solution)**	**Institution where experiment is done**
1.	April 2018 (TFT)	October 17, 2018.	6 months	Center for Electron Microscopy at the Faculty of Biology, University of Belgrade
2.	March 2015 (NW)	June 20, 2019.	4 yr. 3 months	TEM Laboratory Faculty of Agriculture, University of Belgrade
	May 2018 (TFT)		1 yr.1 month	
3.	April 2022 (TFT)	February 24, 2023	8 months	
4.	October 2021 (TFT)	March 20, 2023	1yr. 5 months	LAUS GmbH, Germany
5.	April 2022 (TFT)	March 25, 2023	8 months	NanoLab, Faculty of Mechanical Engineering, University of Belgrade
6.	October 2021(TFT)	April,28,2023	1yr. 6 months	Fraunhofer-Insitut für Silicatforschung ISC, Germany

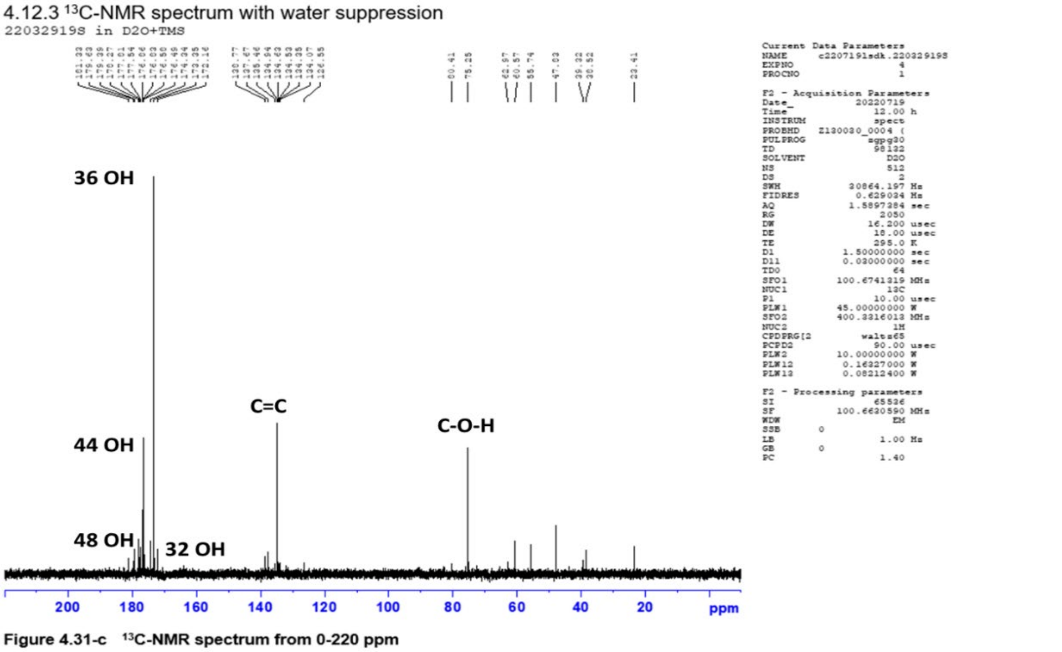

Figure S1. Fullerenol 13C-NMR spectra and OH groups determination (orginal image from device print). Fullerenol 100% - powder, thirteen peaks from 172 to 181 ppm shows different forms of fullerenol (from 30 to 50 OH groups) with dominant peak at 176.49 ppm (36 OH group). The second peaks from 126-138 ppm showed C=C in C_{60}, while peaks 80.41 ppm represent C- OH group in hydroxylated fullerene. Average percentages (%) of OH groups number are: 32 is ~3%, 36 is ~68%, 44 is ~22%, 48 is ~5% and others ~2%. In the paper 36 OH group is considered as average number.

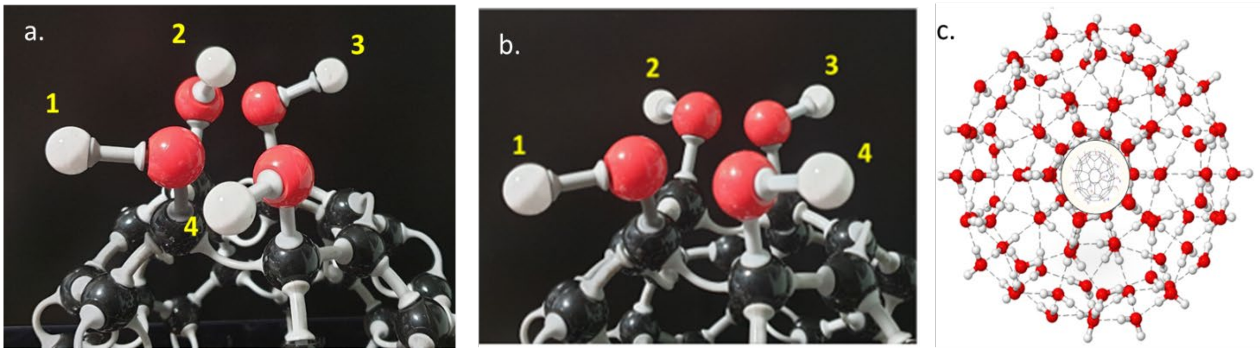

Figure S2. Structure order of fullerenol and 3HFWC. Dilema about order of SD-C_{60} (3HFWC) using two different methods ^{1}H-NMR and XRD. The results of 1H-NMR gave a better arrangement of the structure in SD-C_{60} (3HFWC) than in FD-C_{60} (fullerenol), while the situation with XRD results is the opposite. In order to distinguish between them , we should bear in mind that we used the 1H-NMR technique to identify the positions of hydrogen (protons), while we used the XRD technique to identify the arrangement of the structure in crystal lattices. As can be seen from the pictures (a. and b.), the position of the hydrogen atom (proton) can be different due to the possibility of rotation of the OH group which is connected to carbon. Thus, very different positions can occur for 36 OH groups, while in SD-C_{60} this is not the case, because all hydrogen atoms (protons) are fixed in three-dimensional Penros tiling (3DPT - The Penrose tiling pattern is a type of quasicrystal, which means that it has an ordered yet never-repeating structure), figure c. However, when viewed from the perspective of the XRD method, fullerenol is a molecular crystal with icosahedral symmetry, so its structure is well ordered. As 3DPT are aperiodic structures of the fullerene-water complex (SD-C_{60}), its order is smaller than that of fullerenol.This is similar to the difference between the order of a classical crystal (diamante, crystal lattice clearly defined) and DNA (as aperiodic structures where pairs appear alternately A=T and C≡G). And precisely because SD-C_{60} is not a classically crystallographic ordered structure (the same crystal cell is repeated) but a structure with aperiodic repetition of 3D-Pernos tilings, it possesses special properties that we are only now begin to discover and investigate experimentally .

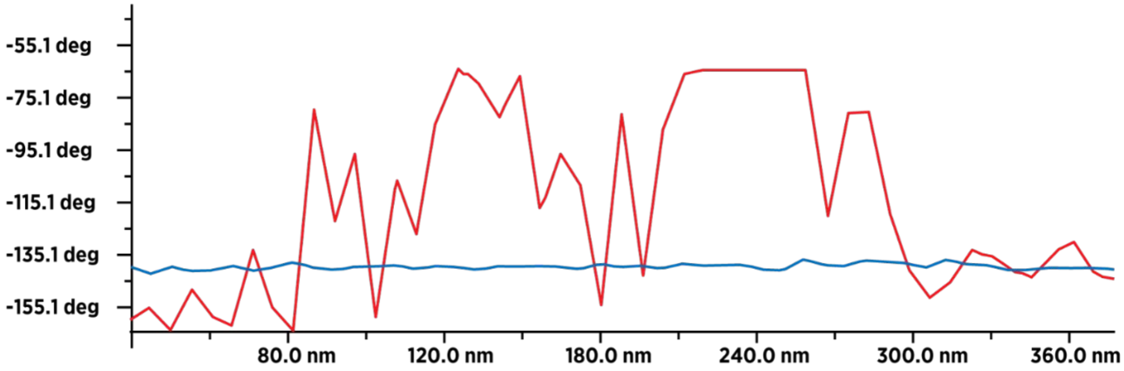

Figure S3. Water presence in fullerenol and 3HFWC. Presence of water molecules in FD-C_{60} (humidity) and SD-C_{60} (humidity + water layers). This is comparative diagrams (from Figure 11 and 12, range 0-400 nm) of intensity values of dipole-dipole interactions between MFM magnetic tip and substrate (FD-C_{60} - blue line and SD-C_{60} - red line). FD-C_{60} has a low intensity of dipole-dipole interaction due to humidity, while SD-C_{60} has a high intensity of dipole-dipole interaction because, in addition to humidity, it contains water molecules packed in layers ,shells (3DPT: three-dimesional Penrose tilies) [22]

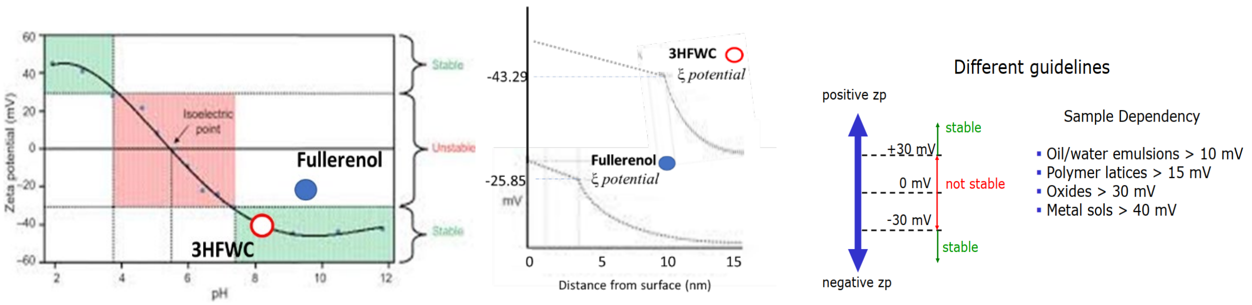

Figure S4. Zeta potential of fullerenol and 3HFWC. Zeta potential of fullerenol and 3HFWC. The figures show the comparative values of zeta potential for fullerenol (-25.85 mV) and 3HFWC (-43.29 mV), as a criterion of stability. It is observed that the 3HFWC substance has almost twice higher the value of the zeta potential then fullerenol, which also points to its greater stability. Also, if we compare the zeta potential of 3HFWC with metal sols, the value is approximately the same. This shows that water layers arranged as a three-dimensional Penrose tiling (3DPT) [22] have special physical properties that give the entire 3HFWC structure stability.

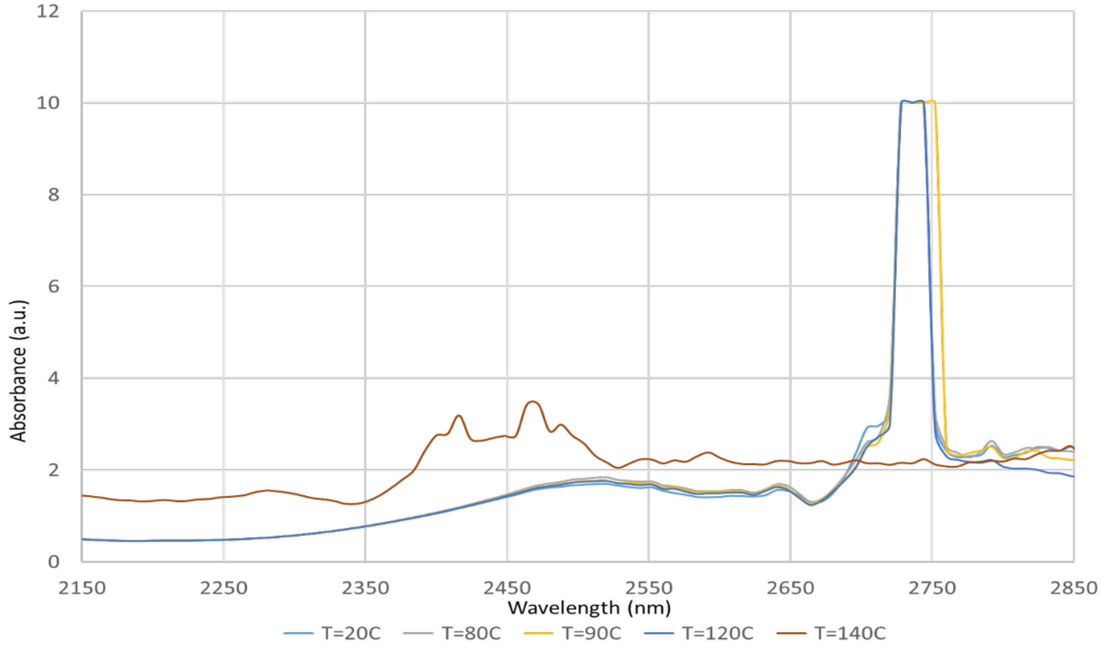

Figure S5. NIR Spectra of 3HFWC for different temperatures. NIR spectra in the domain of hydrogen bonds of 3HFWC (SD-C_{60}) solution with a concentration of 0.150 g/L for temperatures of 20°C , 80°C, 90°C, 120°C and 140°C. As can be seen from the diagram, in the domain of 2,150-2,850 nm, there are no significant differences in the spectrum from 20°C to 120°C, which means that the hydrogen bonds in the water layers of 3HFWC have not been destroyed or lost their intensity. However, for temperature 140°C spectra is different, particularly in domain from 2,700 to 2,800 nm, and it can be seen that the intensity of hydrogen bonds is small and that they are actually broken. At what temperature, between 120°C and 140°C, does evaporation occur? The answer to this question is given by the TGA/DTA experiment, peak at 132.9°C (133°C) with exothermic reaction (Figure 15). At 140°C, the structure of the water layers of SD-C_{60} not only breaks down into water molecules, but the water molecules break down into OH and H, as well as the OH group of fullerenol into O and H. Different structures are formed, resulting in combined vibrations of fragmental structures in the domain of 2,350-2,500 nm, which can be seen in the diagram (red line).
